# Dendrimers and Dendrons as Versatile Building Blocks for the Fabrication of Functional Hydrogels

**DOI:** 10.3390/molecules21040497

**Published:** 2016-04-15

**Authors:** Sadik Kaga, Mehmet Arslan, Rana Sanyal, Amitav Sanyal

**Affiliations:** 1 Department of Chemistry, Bogazici University, Istanbul 34342, Turkey; sadik.kaga@boun.edu.tr; 2 Department of Polymer Engineering, Yalova University, Yalova 77100, Turkey; mehmet.arslan@yalova.edu.tr; 3 Center for Life Sciences and Technologies, Bogazici University, Istanbul, 34342, Turkey

**Keywords:** dendrimers, dendrons, polymers, hydrogels, click chemistry

## Abstract

Hydrogels have emerged as a versatile class of polymeric materials with a wide range of applications in biomedical sciences. The judicious choice of hydrogel precursors allows one to introduce the necessary attributes to these materials that dictate their performance towards intended applications. Traditionally, hydrogels were fabricated using either polymerization of monomers or through crosslinking of polymers. In recent years, dendrimers and dendrons have been employed as well-defined building blocks in these materials. The multivalent and multifunctional nature of dendritic constructs offers advantages in either formulation or the physical and chemical properties of the obtained hydrogels. This review highlights various approaches utilized for the fabrication of hydrogels using well-defined dendrimers, dendrons and their polymeric conjugates. Examples from recent literature are chosen to illustrate the wide variety of hydrogels that have been designed using dendrimer- and dendron-based building blocks for applications, such as sensing, drug delivery and tissue engineering.

## 1. Introduction

**Scheme 1:**
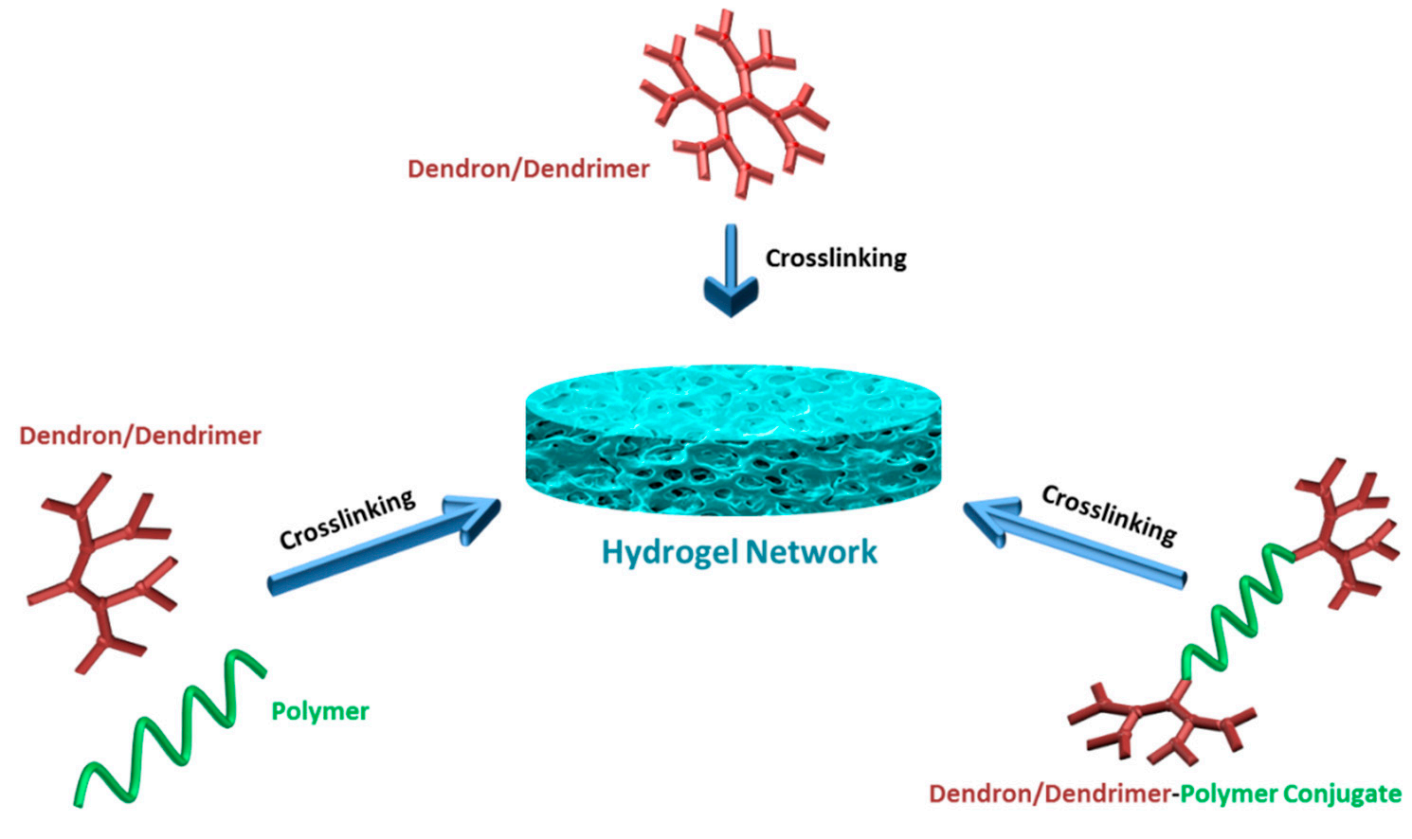
Approaches towards the fabrication of hydrogels using dendron- and dendrimer-based building blocks.

The design and synthesis of novel functional polymeric materials play crucial roles in advancing various fields of biomedical sciences ranging from disease diagnosis to treatment. In recent years, crosslinked polymeric networks, namely hydrogels, have emerged as frontline materials of choice for applications ranging from tissue engineering [1,2] drug delivery [3] and regenerative medicine [4] to biological sensing [5]. Apart from the final physical and chemical properties of hydrogels, the efficiency of their formation at the point of application, under challenging conditions, such as the biological milieu, plays an important role. The branched architecture of dendrimers and dendrons presents a multivalent and multifunctional unit that can be utilized to produce effective interactions through chemical or physical crosslinking (Scheme 1). Furthermore, the design of multivalent polymeric materials, such as dendron/polymer conjugates by appending dendritic structures at chain-ends of polymers, expands the repertoire of hydrogel precursor materials even further. Placement of dendritic reactive units can be envisioned to enhance the number of crosslinking interactions and, hence, to furnish robust materials with high efficiency. The aim of this review is to highlight recent examples of functional hydrogels fabricated using dendritic building blocks and, thus, to stir interest among researchers working in the area of hydrogels to consider the utilization of these materials to address challenging problems in the biomedical arena. This review briefly discusses physically crosslinked hydrogels obtained using only dendritic components, followed by a more detailed discussion on hydrogels derived from a combination of dendrons/dendrimers and polymeric building blocks.

## 2. Physically Crosslinked Hydrogels Fabricated Using Dendritic Building Blocks

Several studies in recent years have demonstrated the utilization of dendron- and dendrimer-based building blocks to mediate the formation of self-assembled nanoscale networks [6]. Hydrophilic gel networks based on dendritic macromolecules have been evaluated as medium-sensitive matrices and as promising delivery systems [7]. In the fabrication of such hydrogel networks, molecules with dendritic architectures serve as low molecular weight hydrogelators, usually resulting in supramolecular hydrogels. For such soft materials, it is often possible to adjust the sol-gel dynamics by changing the reaction conditions. This enables access to metastable gels with tunable mechanical and morphological properties. The gelatin mechanisms of such hydrogels usually include the self-assembly of low molecular weight hydrogelators in water via several non-covalent interactions. The predominant non-covalent intermolecular interactions resulting in physically crosslinked hydrogels include hydrogen bonding, π-π stacking and van der Waals interactions. Recent interest focuses on the design of stimuli-responsive supramolecular hydrogels that can furnish interesting sensing platforms.

**Figure 1:**
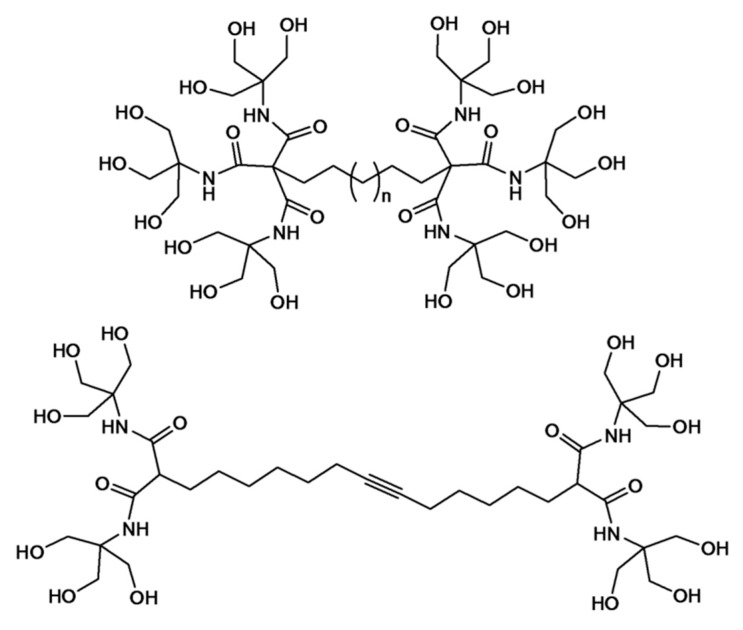
Gel-forming bolaamphiphiles. Adapted with permission from [10], Copyright © 1997 American Chemical Society.

In a seminal contribution, Newkome and co-workers reported the elaboration of dendritic molecules as effective hydrogelators. The so-called Newkome-type arborols were designed as bolaamphiphiles (two-headed amphiphiles) that contain two water-soluble hydroxyl head groups linked through a hydrophobic spacer (Figure 1). Such structures exhibited thermally-reversible aqueous gel formation. The hydrogelation reaction kinetics was shown to be affected by the length of hydrophobic spacer and dendron generation [8]. The gel formation is explained with an aggregation model in which the polar hydroxyl groups provide solubility and the hydrophobic alkyl spacer promotes aggregation. In the case of [9-OH]-10-[9-OH] as a model (containing 9 hydroxyl groups and 10 spacer methylene units) dendrimer, gelation was readily induced when aqueous solutions (2%–10% *w*/*w*) were heated to 80 °C and cooled back to 25 °C. The mechanical and morphological analysis of the resultant gels was demonstrated by various methods, including light scattering, optical microscopy, TEM and viscometry. At the onset of gelation, the viscosity increased rapidly. According to TEM analysis, rod-shaped fibrous aggregates with uniform diameters (34–36 Å), but variable lengths (*ca.* >2000 Å) were observed. This observation was explained by the interlocking network of dumbbell-shaped arborols to maximize the intermolecular lipophilic and hydrophilic interactions resulting in long and thin fiber aggregates. Newkome and co-workers subsequently reported the gel formation of a bolaamphiphile with a central triple bond in the hope of obtaining a polyacetylene covalent structure from pre-organized stacked cores (Figure 1) [9,10]. After gel formation, a novel morphology of a helical supramolecular structure was revealed by electron microscopy. This outcome was attributed to the rigidity of the alkyne units of the arborol that limit the compressibility of alkyl backbone, resulting in non-orthogonal stacking.

**Figure 2:**
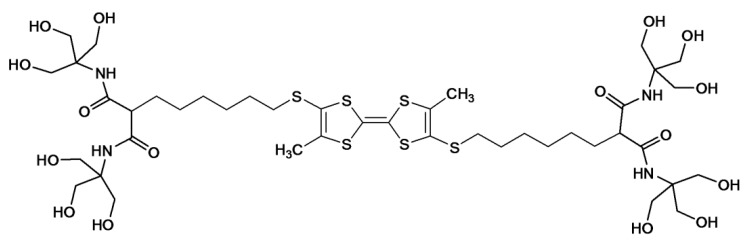
Molecular structure of gel-forming bolaamphiphile with a tetrathiafulvalene spacer. Adapted with permission from [11], Copyright © 1994 American Chemical Society.

The approach of arborol self-assembly in gel synthesis was thereafter exploited by Jorgensen *et al.* using a bolaamphiphile with a tetrathiafulvalene (TTF) spacer (Figure 2) [11]. The TTF structure was installed for electrical conductivity in conjunction with an appropriate dopant, with the aim of constructing a self-assembling molecular wire. Gelation was induced upon cooling a hot solution of the dendrimer in a 25% (*v*/*v*) ethanol–water or DMF–water mixture. Phase contrast optical microscopy and atomic force microscopy (AFM) analyses of the gel revealed string-like superstructures with lengths of the order of microns and diameters ranging from about thirty to several hundred nanometers. After iodine oxidation of the gel, UV-VIS analysis displayed characteristic absorption bands of TTF dimers or oligomers.

Since these early examples, the interest in the synthesis of hydrogels based on dendritic molecules has expanded to include a wide range of structures as potential hydrogelators. As an example, Boons and co-workers evaluated the assembly of a series of glycodendrons in terms of hydrogelation behavior [12]. Glycodendrimers with varying branch lengths between the branching points and the 1,3-dicarboxy-5-hydroxybenzene-based core moiety formed thermally-reversible hydrogels. It was observed that the hydrogelation behavior was significantly altered by structural variations, such as branch length.

**Figure 3:**
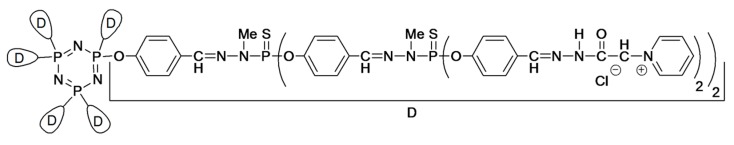
Molecular structure of hydrogelators based on ammonium-functionalized phosphorus-based dendrimers. From [13], Copyright © 2001 by John Wiley & Sons, Inc. Adapted by permission of John Wiley & Sons, Inc.

Another structurally different example capable of gel formation in water includes the surface-modified organophosphorus dendrimers reported by Majoral and co-workers (Figure 3) [13]. The alkylammonium or pyridinium surface-functionalized dendrimers furnished hydrogels through a slow process (heating the solution at 60–65 °C for 11–13 days), but the gelation could be accelerated by using water-soluble additives (e.g., buffers, metal salts, acids, *etc.*). The gelation mechanism was ascribed to the supramolecular interactions, such as hydrogen bonds, π-stacking and hydrophilic/hydrophobic interactions of dendrimer end groups to drive the hydrogelation. The same group reported the formation of hydrogels and macroscopic fibers using water-soluble, poly-cationic phosphorus dendrimers through salt-induced self-assembly [14]. Thermoreversible hydrogels with tunable sol-gel transition temperatures spanning 2–80 °C were shown to be affected by the nature of the salt, dendrimer generation, concentration and temperature.

**Figure 4:**
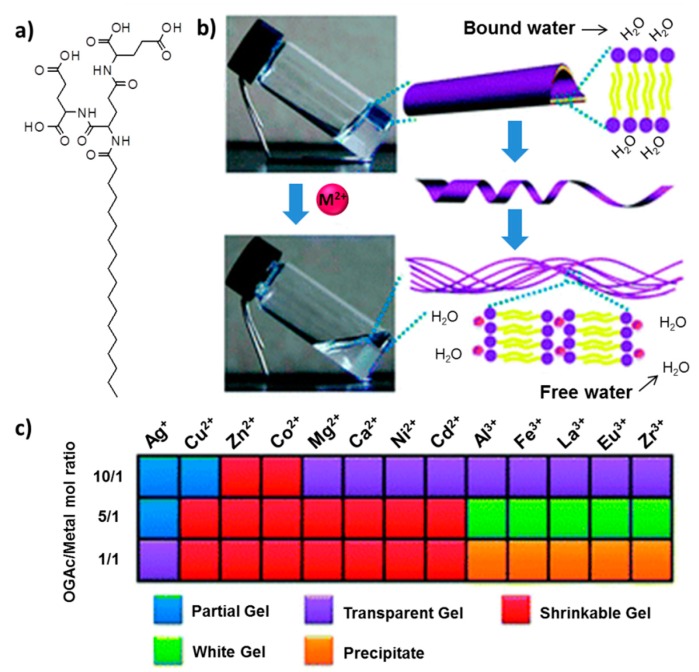
(**a**) Structure of the hydrogelator (OGAc); (**b**) metal ion-triggered shrinkage of the supramolecular hydrogel; (**c**) effect of metal ions on the gelation properties. Adapted from [17] with permission of The Royal Society of Chemistry.

The unique abilities of peptides to form various secondary-, tertiary- and quaternary-level structures provide expedient opportunities in supramolecular hydrogel design that are not easily accomplished with small organic molecules or polymers [15]. Liu and co-workers utilized first- and second-generation l-glutamic acid-based dendrons as peptide-based amphiphilic dendrons that self-assemble into a hydrogel over a wide pH-range [16]. Dendrons with long alkyl cores hierarchically self-assemble into nanofibers or helical nanotube structures over a pH range from 2–13. Interestingly, various chiral nanostructures were obtained upon the regulation of simple pH variations. The same group utilized the peptide-dendron in the fabrication of metal ion-triggered shrinkable supramolecular hydrogel [17]. It was revealed that the presence of certain metal ions (e.g., Mg^2+^, Cu^2+^) can significantly influence the gelation (Figure 4). The critical gelation concentration (CGC) was found to be directly related to the type of metal ions and the ratio of ions. Hydrogels containing divalent metal ions exhibited continuous shrinkage. This shrinkage is a reversible phase transition, and upon heating of the shrunken gel, the phase turns into a sol, and then, the gel is reformed. The reversibly-shrinkable hydrogel was employed in controlled release of vitamin B1 (VB_1_). It was shown that, after the addition of divalent ions, the hydrogel allowed the release of VB_1_, and the release kinetics was accelerated by hydrogel shrinkage. These shrinkable metallo-hydrogels also displayed reversible volume-phase transitions regulated by pH change [18].

**Figure 5:**
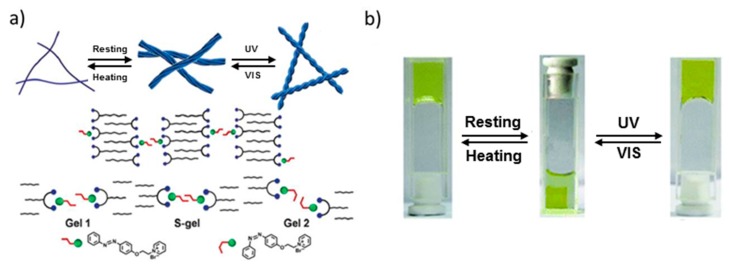
(**a**) Schematic illustration of hierarchical co-assembly by amphiphilic dendron and azobenzene components through electrostatic interaction; (**b**) thermal and photo-switched reversible control on network composition. Adapted from [20] with permission of The Royal Society of Chemistry.

Another class of stimuli-responsive hydrogel systems, a photo-responsive low molecular weight hydrogelator based on a dendron with an anthracene core and gluconamide peripheral units, was reported by Sako and Takaguchi [19]. Photo-responsive hydrogels undergo macroscopic volume phase transitions or shrinking/swelling due to the photoisomerization of reactive chromophores in the gel matrix. The anthracene moieties were shown to undergo photodimerization upon photoirradiation (λ > 300 nm), resulting in a gel-to-sol transition of the hydrogel. Another photo-responsive system was recently reported by Liu and co-workers by employing co-assembly of an l-glutamic acid-terminated amphiphilic dendron and a positively-charged azobenzene [20]. When these two components are mixed, an initial gel phase is obtained resulting from the multi-bilayer structure through the electrostatic interaction of the components (Figure 5a). The gel matrix undergoes shrinkage upon resting at room temperature due to the aggregation of azobenzene moieties. The UV photo-irradiation of the shrunken gel allows cis-trans isomerization of the azobenzene groups, thus allowing the expansion of distances between layers, resulting in swelling of the hydrogel. This mechanism provides both thermal and photo-switchable control on network structure and swelling/deswelling kinetics (Figure 5b).

**Figure 6:**
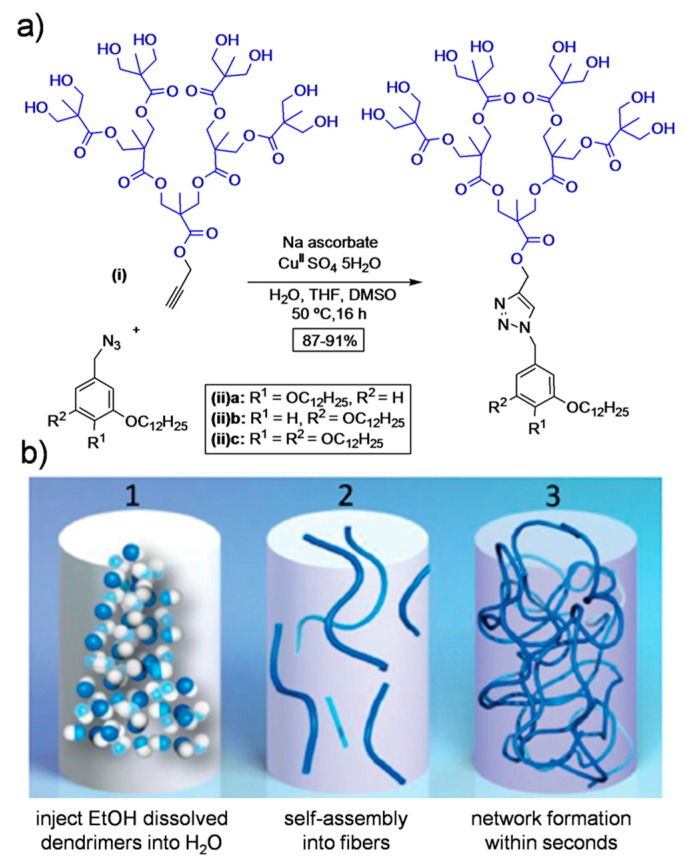
(**a**) Structure and synthesis of amphiphilic Janus dendrimers; (**b**) hierarchical self-assembly of dendrimers to form fibers and hydrogel. From [21], Copyright © 2015 by John Wiley & Sons, Inc. Adapted by permission of John Wiley & Sons, Inc.

Recently, Nummelin *et al.* demonstrated the synthesis of supramolecular hydrogels based on amphiphilic Janus dendrimers containing *O*-alkylated benzyls as the hydrophobic segment and a hydroxyl-terminated 2,2-bis(-methylol)propionic acid (bis-MPA)-based hydrophilic segment [21]. The target dendrimers were synthesized by utilizing copper-catalyzed azide-alkyne click chemistry (Figure 6). These dendrimers formed hierarchical fibrous architectures in aqueous solution leading to large fibers and hydrogels. The storage moduli and melting temperature of hydrogels were shown to be regulated by the position or number of hydrophobic alkyl chains in the structure. The thus fabricated hydrogels were employed in the controlled release of nadolol (small-molecule drug), gonadorelin (decapeptide) and HRP (enzyme).

## 3. Fabrication of Hydrogels Using Dendrimers and Dendrons as Building Blocks Using Polymeric Crosslinkers

Generally, hydrogels used in various biomedical applications involve chemical crosslinking, since they are robust and can maintain their stability in the biological environment. In this part of the review, we focus on the fabrication of functional hydrogels through crosslinking of dendrimers and dendron-containing building blocks using “click” reactions, chemical ligations and other alternative chemistries.

**Figure 7:**
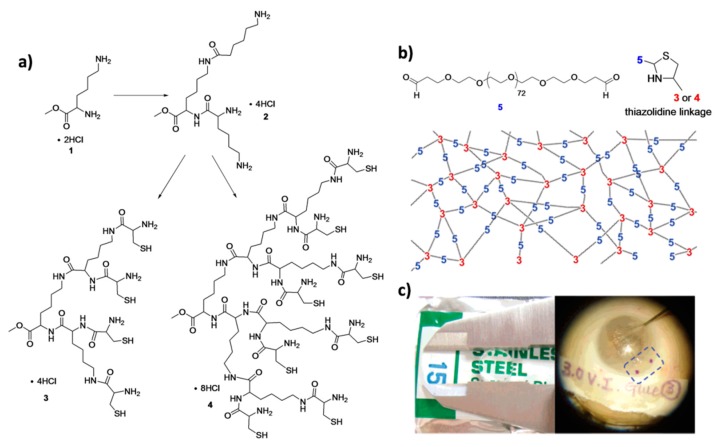
(**a**) Synthesis of peptide dendrons; (**b**) structure of macromers and the thiazolidine linkage; (**c**) synthesized hydrogel (left) and hydrogel sealant-repaired corneal incision. Adapted with permission from [22], Copyright © 2004 American Chemical Society.

Hydrogel-based sealants for small corneal incisions after cataract operations were fabricated by Grinstaff and co-workers using native chemical ligation between peptide-based dendrons and linear poly(ethylene glycol) (PEG). *In situ* crosslinked hydrogel was formed by chemical ligation between cysteine-containing peptide dendrons and homobifunctional PEG-bearing propionaldehyde (P-Ald PEG) [22]. Lysine-based peptide dendrons with four or eight N-terminal cysteine groups were synthesized. Upon mixing the aldehyde containing PEG with the dendrons, a thiazolidine linkage was formed between cysteine and aldehyde groups under mild aqueous conditions (in HEPES buffer, pH: 7.4) to yield hydrogels within a few minutes (Figure 7). They measured the mechanical properties and the refractive index values of cylindrical hydrogels prepared by different dendron generations and macromer concentrations. The *ex vivo* results on enucleated eyes showed that hydrogels possess better parameters for application compared to suture and unassisted healing. They improved sealing properties and reduced potential complications.

**Figure 8:**
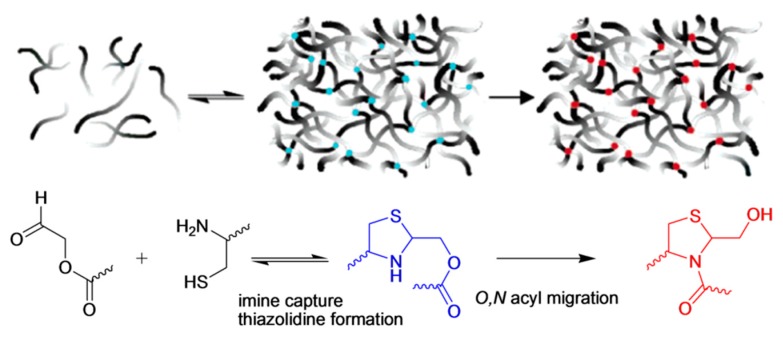
Schematic illustration of hydrogel formation (**top**) and pseudoproline ligation for hydrogel formation (**bottom**). Reprinted with permission from [23], Copyright © 2006 American Chemical Society.

As an extension of this work, Grinstaff and co-workers employed pseudoproline formation toward the construction of these hydrogels to accelerate the crosslinking and to prolong their shape stability [23]. The same peptide-based dendrons were used, but instead of P-Ald PEG, PEG-ester aldehyde (E-Ald PEG) was utilized for the thiazolidine formation. The reversible nature of the thiazolidine bond and ester group adjacent to the aldehyde group led to the formation of an irreversible pseudoproline linkage via O,N acyl migration (Figure 8). These hydrogels were formed in 30 s upon employing E-Ald PEG. Moreover, the hydrogels prepared by E-Ald PEG remained stable for more than six months with a small weight change (less than 10%), whereas the hydrogels prepared by P-Ald PEG had lost more than 50% of their initial weight in six days. *In vitro* results on an enucleated eye showed that these hydrogels reduced the number of sutures required for securing corneal transplants from 16 to eight, but could not secure the incision by itself due to insufficient adhesivity.

Grinstaff and co-workers also performed a comparative study for better understanding of the adhesive, physical and mechanical properties of hydrogels formed by peptide-based dendrons and P-Ald PEG, E-Ald PEG and a homobifunctional PEG-bearing butyraldehyde (B-Ald PEG) [24]. They obtained tunable mechanical properties by changing the polymer and gelation concentrations. The degradation times of hydrogels prepared by P-Ald PEG, B-Ald PEG and E-Ald PEG were six days, 1.5 and 24 weeks, respectively. The best adhesive behavior was obtained with B-Ald hydrogel on an enucleated eye model. This study highlighted that materials with a wide range of mechanical properties and degradation times and sufficient adhesiveness can be prepared, and these hydrogels would be useful as wound-healing scaffolds for applications requiring different healing times.

**Figure 9:**
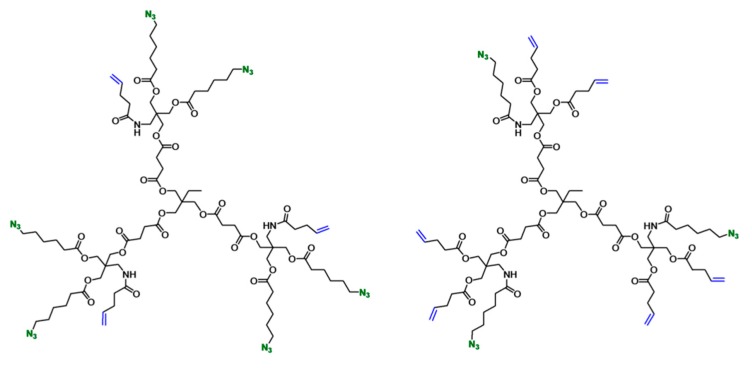
Polyester dendritic scaffolds displaying three alkene and six azide groups (**left**) and six alkene and three azide groups (**right**). Adapted from [25], published by The Royal Society of Chemistry.

Since the advent of “click” reactions, several of these efficient transformations, such as the thiol-ene and strain-promoted azide-alkyne cycloaddition, have been used for *in situ* hydrogel formation using dendrimers and functional polymers. In a recent study, Malkoch and co-workers synthesized orthogonally-functionalized dendrimers and used them with PEG dithiol to obtain functional hydrogels [25]. They designed two sets of polyester dendritic scaffolds, the first one displaying three alkene and six azide groups and the second one with six alkene and three azide groups (Figure 9).

The alkene groups were used for crosslinking with PEG dithiol (M_w_ = 6 kDa) to yield hydrogels via a UV-initiated thiol-ene click reaction. Selective functionalization of these dendritic scaffolds was demonstrated by modification with D-mannose, L-DOPA and carboxylic acid-bearing alkyne groups via the Huisgen-type copper-catalyzed azide-alkyne cycloaddition (CuAAC) reaction. These hetero-bifunctional dendrimers were also evaluated as promising candidates for fracture stabilizing bone adhesives.

A rapid hydrogel formation was accomplished using a thiol-ene reaction between a poly(amido amine) (PAMAM) dendrimer containing vinyl sulfone groups on its periphery and thiolated hyaluronic acid by Wang and co-workers for biofabrication applications [26]. A generation 5 (G5) PAMAM dendrimer possessing 75 acetyl and the 35 residual amine groups were reacted with Traut’s reagent to obtain thiol functional groups. Thiol groups on the periphery of the dendrimer were reacted with divinyl sulfone (DVS) to install vinyl sulfone groups. After partial modification of vinyl sulfone groups with arginine-glycine-aspartic acid (RGD), they obtained the G5 PAMAM Ac (75)-DVS (30)-RGD (5) dendrimer. Hydrogels were obtained by mixing this dendrimer with thiolated hyaluronic acid. They showed that the viability, proliferation and attachment of rat bone marrow stem cells in the *in situ*-formed hydrogels were improved upon RDG modification.

Gitsov and co-workers reported amphiphilic hydrogel formation using crosslinking between isocyanate or epoxy-terminated telechelic PEGs (M_w_ = 3400 g/mol) and poly(benzyl ether) dendrimers containing peripheral amine groups [27]. Fast hydrogel formation was observed in the case of PEG diisocyanate due to the high reactivity of isocyanate groups towards amines. They reported that this fast crosslinking resulted in physical entanglement of PEG chains and low crosslink density. However, using diepoxy PEG, highly-crosslinked networks were produced due to the stability of epoxides under gelation conditions. They demonstrated that the swelling properties of these hydrogels were influenced by the PEG content and dendrimer generation. In their following study, the group investigated these hydrogels in terms of their environmental response and feasibility for bioanalytical and biomedical applications [28]. Likewise, Hedden and Unal used amine-terminated PAMAM dendrimers and epoxide-terminated telechelic PEG (M_w_ = 4000 g/mol) for the construction of hydrogels [29]. Hydrogels were formed using generation 0 (G0), generation 2 (G2) and generation 4 (G4) PAMAM dendrimers with different polymer concentrations and dendrimer/PEG mole ratios to understand the effect of these modifications on gelation and swelling properties. Hydrogels showed a wide range of swelling ratios (from 15–1000), where the swelling behavior was mostly affected by the stoichiometric ratio between amines and epoxides. They demonstrated that irrespective of the dendrimer generation, superabsorbent hydrogels can be achieved using a large excess of dendrimers over PEG. The same group reported another study on pH-dependent swelling of these hydrogels [30].

**Figure 10:**
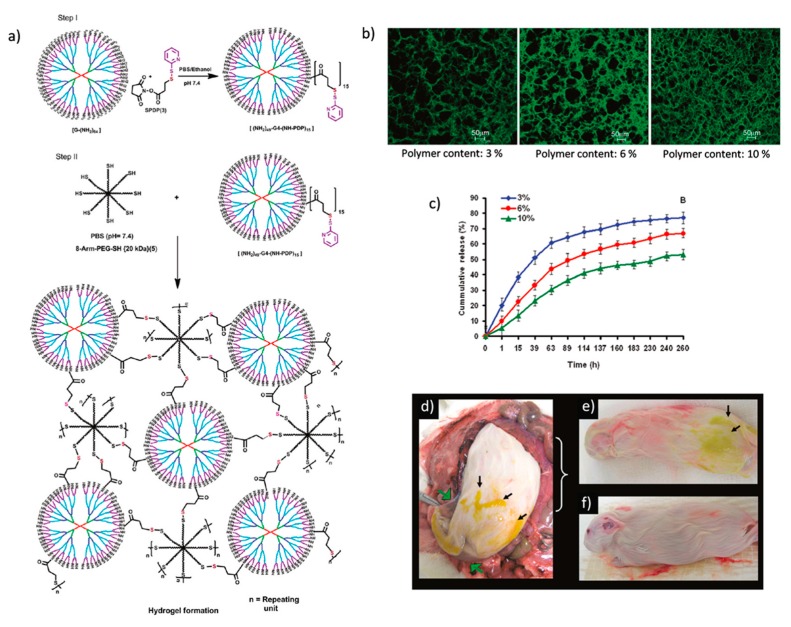
(**a**) Disulfide formation for crosslinking; (**b**) 3% hydrogel, 6% hydrogel and 10% hydrogel show the crosslinking network changes; (**c**) cumulative release profiles of hydrogels; degraded hydrogel on fetal membrane; (**d**,**e**) no signs of transfer inside the fetal membrane; (**f**) adapted with permission from [31], Copyright © 2011 American Chemical Society.

Alternative strategies, such as the pyridyl disulfide-thiol exchange reaction and enzymatic reactions, have been used for hydrogel formation using dendrimers along with polymers as building blocks. In a very well-designed study, Kannan and co-workers fabricated biodegradable hydrogels for sustained drug release for treating genital infections [31]. The hydrogels were derived from two components, a pyridyl disulfide (PDS)-appended G4 PAMAM dendrimer and an eight-arm PEG containing peripheral thiols (Figure 10a). They prepared hydrogels in 10–30 s by mixing equal volumes of dendrimer and PEG solutions. Fluorescence microscopy images showed that the pore size in the hydrogel network decreased, and crosslinking density increased by increasing the polymer content (Figure 10b).

The time for the equilibrium swelling state of hydrogels and drug loading efficiency also decreased with the increase in polymer content due to higher crosslinking density. They prepared drug loaded hydrogels by dissolving amoxicillin in an eight-arm PEG solution and mixing this solution with dendrimers to obtain the hydrogels in order to study *in vitro* drug release and perform *in vivo* experiments in a pregnant guinea pig model. Cumulative drug release increased upon the decrease in crosslinking density, and sustained release for more than 240 h was achieved in the *in vitro* experiments. (Figure 10c). They reported that the degradation of hydrogels started after 72 h. The *in vivo* experiments showed that degraded hydrogels were detained in the maternal tissues (Figure 10d,e) and did not transfer across the fetal membrane (Figure 10f). They concluded that localized selective treatment can be achieved for genital infections during pregnancy without affecting the fetus using these hydrogels.

As a different approach, Tran and co-workers reported the use of an enzymatic reaction to obtain biocompatible hydrogels from cationic PAMAM dendrimers and tetronic, a non-toxic surfactant [32]. The G3 PAMAM dendrimer (Den) was modified with p-hydroxyphenyl acetic acid (HPA) via carbodiimide coupling to obtain Den-HPA. Tyramine modification of tetronic (TTe) was accomplished by activation of terminal hydroxyl groups via nitrophenyl chloroformate and tyramine substitution. Hydrogel formation was accomplished through the reaction between phenol groups of Den-HPA and TTe, catalyzed by horseradish peroxidase enzyme (HRP) and hydrogen peroxide. Heparin-loaded hydrogels formed by changing the Den-HPA/TTe ratio showed better sustained heparin release (over one month) profiles compared to heparin-loaded TTe hydrogel, and an increase in dendrimer content resulted in lower cumulative heparin release.

Zhuo and co-workers designed physically-crosslinked hydrogels based on poly(vinyl alcohol) (PVA) and amine-terminated G6 PAMAM dendrimers [33]. The purpose was to improve the release and drug loading properties of PVA hydrogels for small drug molecules by introducing the PAMAM dendrimer. Physical crosslinking was achieved by hydrogen bonds among hydroxyl groups of PVA and amine and the amide groups of the PAMAM dendrimer by the process of freeze/thaw cycles. They reported that increasing the number of freeze/thaw cycles enhanced the formation of polymer crystallites expressing the hydrogen bonds. They prepared hydrogels by tailoring the PAMAM dendrimer content in hydrogel precursors and demonstrated that the increase in dendrimer content resulted in a faster swelling rate and higher swelling ratios.

## 4. Fabrication of Hydrogels Using Polymer Conjugates of Dendrimers and Dendrons as Building Blocks

**Figure 11:**
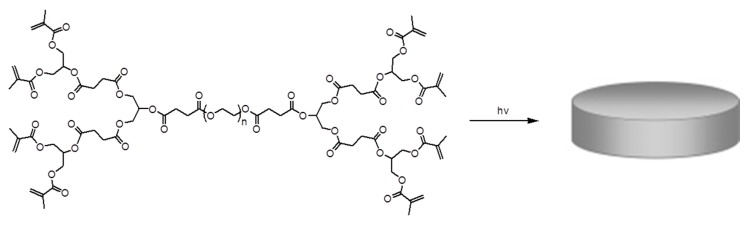
Photo-crosslinkable methacrylated generation 3 (G3) dendrimer PEG ester conjugate. Adapted with permission from [36], Copyright 2006 American Chemical Society.

In last part of the review, we focus on the fabrication of hydrogels using polymeric conjugates of dendrons/dendrimers using various crosslinking strategies. Grinstaff and co-workers used dendritic units composed of biocompatible compounds, glycerol and succinic acid to synthesize tri-block (dendron-linear polymer-dendron = DLD) copolymers [34,35]. Copolymers were designed with poly(ethylene glycol) (PEG) (M_w_ = of 3400 g/moL) as the inner block and dendrons as the outer blocks. They synthesized copolymers containing G0 up to G4 dendrons using the divergent approach. The peripheries of the copolymers were methacrylated for photo-crosslinking. *In situ* dendritic gel sealants were obtained for corneal wound repair using these methacrylated dendron-polymer conjugates with an eosin-Y photo-initiation system, including n-vinyl pyrrolidinone and triethanolamine under argon ion laser irradiation. It was observed that the dendron generation of the copolymer has a significant effect on the crosslinking rate and solubility of copolymers. The copolymer with the G1 dendron showed better sealing behavior among the tested copolymers. In another study, they used the same DLD copolymer with the G1 dendron to synthesize a novel hydrogel for cartilage repair [36]. Methacrylate functionalities at the dendron periphery allowed biocompatible photo-crosslinking with the eosin-Y-based photo-initiation system (Figure 11). They obtained a series of hydrogels with different mechanical properties and degrees of swelling at different gelation concentrations. These showed very small volume changes during *in situ* crosslinking at the cartilage trauma site, which could be due to multivalent functionalities at the periphery of the copolymer, leading to higher crosslinking.

**Figure 12:**
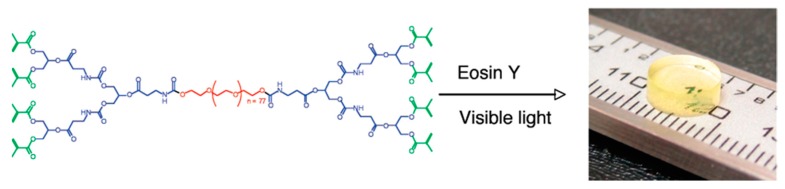
Photo-crosslinkable methacrylated G1 PEG DLD copolymer carbamate conjugate (**left**) and the obtained hydrogel (**right**). Reprinted with permission from [37], Copyright © 2008 American Chemical Society.

In a following study, the same group synthesized a small library of DLD copolymers by an iterative approach using the copolymers previously described with some differences [37]. They used glycerol (GL) together with one or both of the other two biocompatible compounds, succinic acid (SA) and β-alanine (BA) to obtain first-generation dendrimers divergently from PEG, followed by installing methacrylate (MA) groups at the periphery of these copolymers. Hydrogels were obtained using copolymers composed of glycerol (GL) and β-alanine (BA) ([G1]-PGLBA-MA)_2_-PEG), glycerol and succinic acid (SA) ([G1]-PGLSA-MA)_2_-PEG), glycerol, β-alanine and succinic acid ([G0]-PGLBA-[G1]-PGLSA-MA)_2_-PEG and [G0]-PGLSA-[G1]-PGLBA-MA)_2_-PEG) with different concentrations by an eosin-Y-based photo-initiation system (Figure 12). While all hydrogels possessed low swelling characteristics, they displayed different mechanical properties due to differences in their structure, generation size and concentration. They observed that the [G1]-PGLBA-MA)_2_-PEG based hydrogel prepared by the concentration of 20 *w*/*v*% displayed similar stiffness and viscoelasticity parameters with native articular cartilage. They used this hydrogel for a pilot study to test the cartilage repair on damaged rabbit knee and obtained encouraging results.

The same group designed hydrogel-based double-layered corneal implants by sequential injection of gel components in the same mold to increase the adhesion properties of hydrogels [38]. A 2-hydroxyethylmethacrylate and methacrylic acid (HEMA-*co*-MAA)-based hydrogel was used as the top layer, and the DLD dendron-polymer conjugate composed of glycerol and succinic acid based hydrogel was used as the bottom layer of the implant. They covalently modified (HEMA-*co*-MAA) hydrogels with type I collagen for corneal epithelial cell adhesion and modified the dendrimer-based hydrogels with arginine-glycine-aspartic acid (RGD) for corneal fibroblast adhesion. The crosslinked structure of the hydrogel network was obtained using ammonium persulfate-tetramethylethylenediamine (APS-TEMED)-based initiation in the presence of arginine-glycine-aspartic acid-cysteine (RGDC)-PEG-acrylamide to yield an RGD-modified hydrogel. They obtained tunable physical properties by changing the hydrogel solution concentration for double-layered corneal implants. This approach provides a judicious strategy for epithelialization of implanted material and integration into tissue.

**Figure 13:**
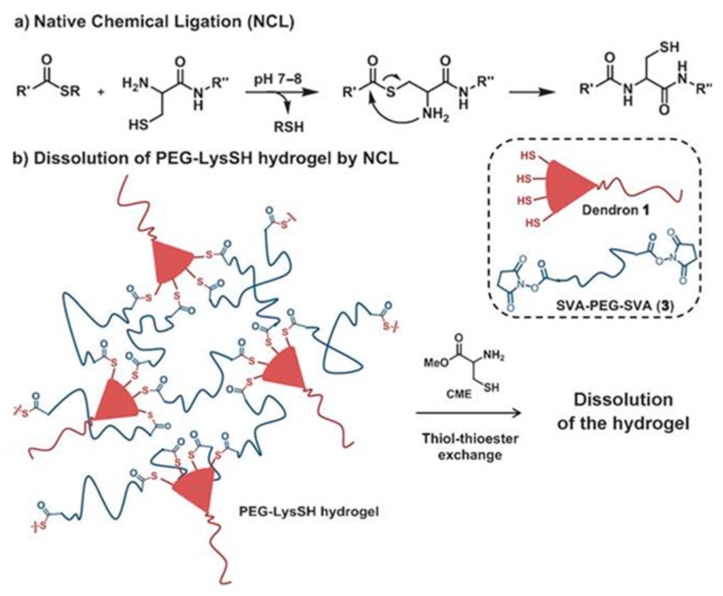
(**a**) Native chemical ligation; (**b**) dissolution of the hydrogel via thiol-thioester exchange (CME: L-cysteine methyl ester). From [39], Copyright © 2013 by John Wiley & Sons, Inc. Reprinted by permission of John Wiley & Sons, Inc.

Besides these photoreactive and APS-TEMED initiations, Grinstaff and co-workers also used amide and thioester linkages for crosslinking of peptide-based dendron polymer conjugates with PEG disuccinimidyl valerate (SVA-PEG-SVA) for functional hydrogel formations (Figure 13) [39]. A dendron-polymer conjugate containing four terminal amines was synthesized (PEG-Lys-NH_2_) and used for the preparation of amide-linked hydrogels as the control material to understand the dissolution mechanism. This dendron-polymer conjugate was elaborated to contain four peripheral SH (thiol) groups (PEG-Lys-SH, Dendron 1). Conjugates possessing four terminal amines and thiols were mixed with a solution of PEG disuccinimidyl valerate (M_w_ = 3400 g/mol), and hydrogels were formed via amide or thioester linkages, respectively, within seconds. Thereafter, they utilized the thiol-thioester exchange chemistry for the dissolution of the prepared hydrogel.

**Figure 14:**
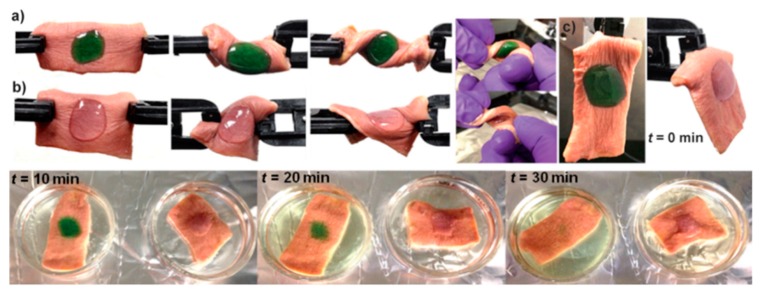
(**a**) Mechanical resistance of hydrogels formed by the thio-ester (green) bond; (**b**) mechanical resistance of hydrogels formed by the amide (pink) bond; (**c**) degradation of the hydrogels in the solution of l-cysteine methyl ester. From [39], Copyright © 2013 by John Wiley & Sons, Inc. Reprinted by permission of John Wiley & Sons, Inc.

*Ex vivo* experiments were performed to understand the adherence and dissolubility of these hydrogels on human skin tissues. The intact form of the hydrogels remained stable after the application of mechanical stress (Figure 14a,b). A solution of l-cysteine methyl ester was used as a nucleophile for thiol-thioester exchange chemistry for disassembly. The authors showed that the hydrogel formed via the thioester bond was completely degraded in 30 min, whereas the hydrogel formed via the amide bond was stable for hours (Figure 14c). They obtained similar results with the closure of a 2.5-mm hole of an *ex vivo* bovine jugular vein under pressure higher than normal blood pressure. The study underlines the importance of such material in cases requiring emergency intervention and subsequent operation.

**Figure 15:**
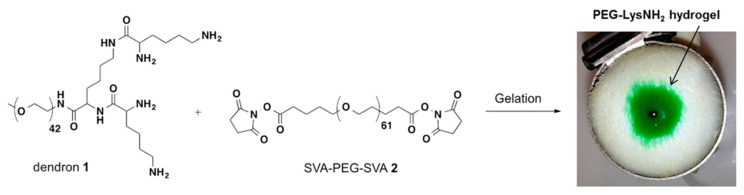
*In situ* formation of PEG-Lys-NH_2_-based hydrogel for sealing small corneal incisions. Reproduced from [40]; published by the BioMed Central.

In a more recent report, Grinstaff and co-workers compared a dendron-polymer (PEG-Lys-NH_2_)-based hydrogel with a commercially available tissue sealant and adhesive products (Figure 15) [40]. The results obtained by *ex vivo* bovine aorta or murine skin tissue samples showed that this material showed the same performance as the tested products when sealing small incisions.

**Figure 16:**
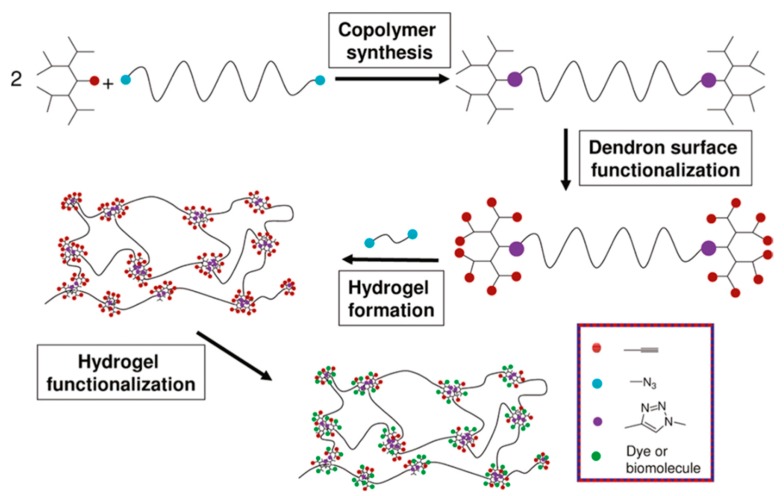
Schematic illustration of dendron polymer conjugate synthesis and hydrogel formation. Reprinted with permission from [42], Copyright © 2010 American Chemical Society.

Since the advent of “click” chemistry, many research groups have employed reactions, such as the thiol-ene, thiol-yne and CuAAC reactions, towards the formation and functionalization of hydrogels [41]. In 2010, Sanyal and co-workers reported a class of functionalizable hydrogels based on dendron-polymer conjugates obtained using polyester dendrons and linear PEG diazides (Figure 16) [42]. Triblock copolymers with second and third generation bis-MPA-based polyester dendrons [43,44] were obtained by coupling dendrons containing an alkyne group at their focal point with PEG diazides via the CuAAC reaction. The peripheral hydroxyl groups on the dendrons were reacted with 4-pentynoic anhydride to install alkyne functionalities at their periphery. Hydrogels were prepared by crosslinking the alkyne terminated dendron-polymer conjugate with tetra (ethylene glycol) diazide using the CuAAC reaction. Functionalizable hydrogels with a tunable degree of residual alkyne groups were obtained by varying the ratio of the alkyne to azide groups in the gel precursor formulation. Controlled crosslinking and functionalization were proven by covalent attachment of various fluorescent dyes, as well by tailored immobilization of streptavidin after controlled attachment of biotin azide onto these hydrogels.

**Figure 17:**
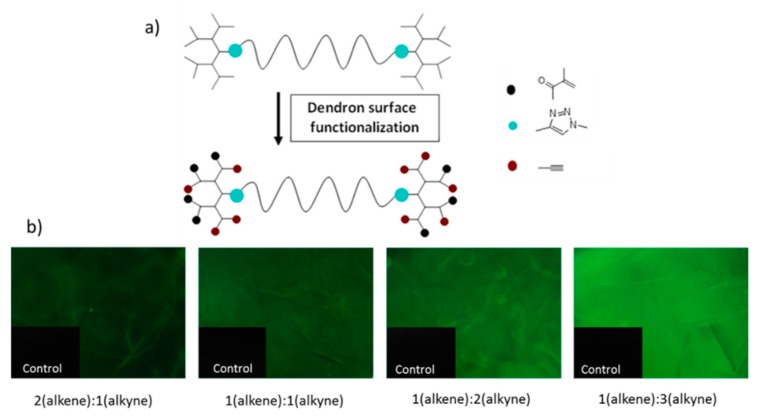
(**a**) Dendron surface modification; (**b**) fluorescent microscope images of obtained bulk hydrogels with different alkene/alkyne ratios after bio-immobilization with biotin azide and FITC-labeled streptavidin interaction. Adapted with permission from [45], Copyright © 2015 American Chemical Society.

In an alternative approach, Sanyal and co-workers recently reported the fabrication of similar hydrogels using orthogonally-functionalizable polyester dendrons [45]. Instead of using the CuAAC reaction for crosslinking, the hydrogels were obtained through photo-crosslinking of the dendron-polymer conjugate. The periphery of the dendrons on the dendron-polymer conjugates were decorated with methacrylate groups for photo-crosslinking and alkyne groups for post-gelation functionalization. Stoichiometric adjustment of the orthogonally-reactive groups on the dendron peripheries provided a tunable degree of crosslinking, as well as alkyne functionalities (Figure 17). Successful covalent modification of bulk hydrogels and hydrogel micro-patterns with control over the extent of functionalization were demonstrated by the attachment of a fluorescent dye, BODIPY azide. Likewise, it was possible to tailor the amount of attached biotin, a bioactive ligand, and, thus, to dictate the extent of the immobilization of streptavidin. Through fluorescence microscopy analysis of functionalized hydrogels, it was concluded that a tailored increase in the alkyne units on the dendrons allows one to attach more biotin azide ligand onto the hydrogels and, thus, to dictate the extent of immobilization of the FITC-labelled streptavidin. This approach allows the decoration of scaffolds with biomolecules with a fair degree of control, an approach that may be relevant in developing tissue engineering or sensing platforms.

**Figure 18:**
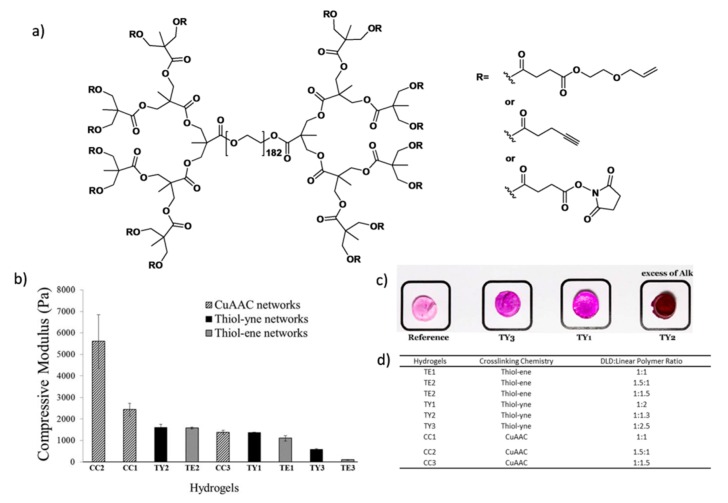
(**a**) Allyl, acetylene or *N*-hydroxy succinimide (NHS) functional dendron polymer conjugates; (**b**) compressive modulus values of the obtained hydrogels; (**c**) post-gelation functionalization of thiol-yne (TY) hydrogels with azide functional rhodamine (reference sample prepared by TY1 hydrogel without Cu(II)SO_4_ catalyst); (**d**) definition of hydrogels. From [46], Copyright © 2015 by John Wiley & Sons, Inc. Adapted by permission of John Wiley & Sons, Inc. CuAAC, copper-catalyzed azide-alkyne cycloaddition.

More recently, Malkoch and co-workers employed various homofunctional dendron-polymer conjugates and crosslinking methodologies for designing hydrogels with different properties for several applications [46]. They prepared dendron-linear polymer-dendron (DLD) conjugates by a divergent approach using PEG diol and a bis-MPA dendron to obtain third-generation polyester dendrons. Then, they functionalized the periphery of the dendrons with allyl, alkynyl and *N*-hydroxy succinimide (NHS) groups (Figure 18a). Hydrogels were obtained using thiol-ene, thiol-yne, CuAAC or NHS ester coupling chemistries with complementary telechelic PEGs. In this study, tunable mechanical properties were obtained by varying the ratio between DLDs and linear PEGs (Figure 18b). Functionalization of the hydrogels was demonstrated through conjugation of an azide-containing rhodamine dye with the residual alkynes groups on the thiol-yne (TY) hydrogels via CuAAC reaction (Figure 18c).

**Figure 19:**
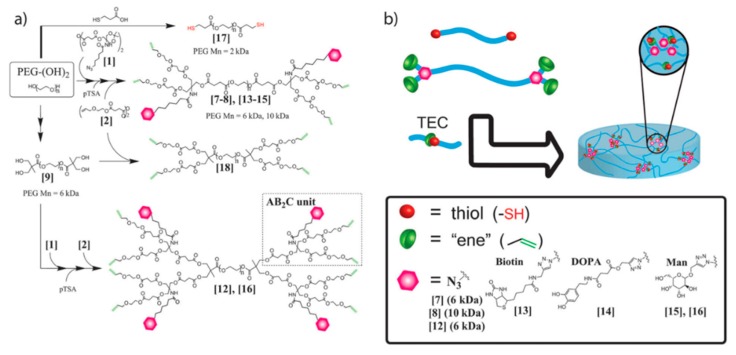
(**a**) Building blocks of hydrogel; (**b**) scheme for hydrogel formation. Adapted from [47]; published by The Royal Society of Chemistry.

Malkoch and co-workers employed UV-initiated thiol-ene click chemistry (TEC) for the synthesis of non-toxic and degradable hydrogels using orthogonally-functionalized dendron polymer conjugates [47]. They used AB_2_C-type monomer for the synthesis of orthogonal polyester dendrons on PEG diols to obtain triblock copolymers (Figure 19). The crosslinking was performed by UV-initiated thiol-ene chemistry between alkenes on the dendron surface and the thiol groups on a linear PEG 2-kDa dithiol. The orthogonally-reactive azide groups on the dendron surface remained for conjugation of other biorelevant moieties, such as an alkyne bearing biotin, l-dopamine and d-mannose using the CuAAC reaction. The authors proposed the feasibility of using these non-toxic hydrogels as an artificial extra cellular matrix for the recovery of damaged tissue.

**Figure 20:**
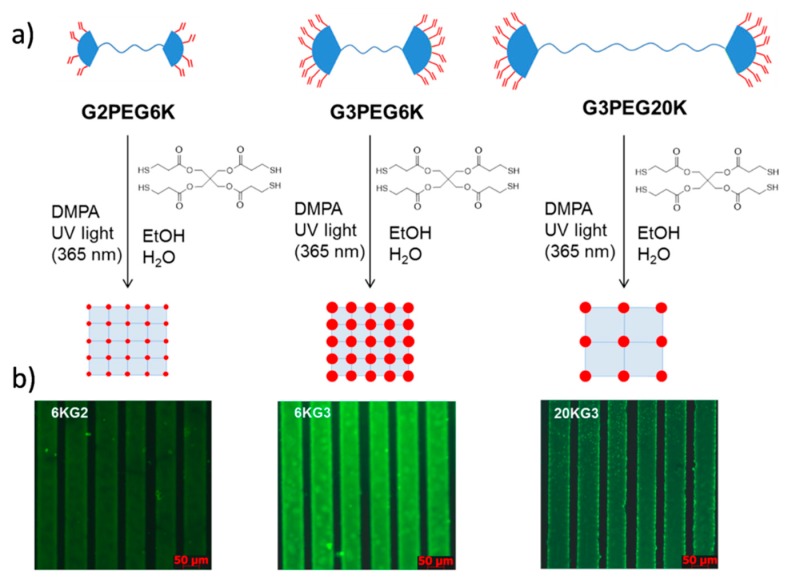
(**a**) Hydrogel formation using dendron polymer conjugates having different PEG lengths and dendron generations; (**b**) fluorescent microscope images of the obtained micropatterned hydrogels after post-modification functionalization with BODIPY thiol (DMPA: 2,2-Dimethoxy-2-phenylacetophenone). From [48], Copyright © 2015 by John Wiley & Sons, Inc. Adapted by permission of John Wiley & Sons, Inc.

Similar to the abovementioned approach by Malkoch and co-workers, Sanyal and co-workers investigated hydrogels using crosslinking of similar dendron-polymer conjugates via the thiol-ene reaction [48]. The dendron-polymer conjugate was synthesized through CuAAC coupling of PEG-diazides with orthogonally-functionalizable dendrons containing an alkyne group at the focal point and alkene groups at their periphery. Hydrogels were fabricated by thiol-ene chemistry between the alkene groups on the dendron-polymer conjugates with a tetrathiol-based crosslinker, pentaerythritol tetrakis (3-mercaptopropionate) (PETMP). Clear and transparent hydrogels were obtained and further functionalized with thiol-containing dye in a sequential manner. They tuned alkene-thiol stoichiometry to obtain residual alkene groups as handles for subsequent functionalization on bulk and micropatterned hydrogels (Figure 20). The tunability of water uptake and the degree of functionalization was obtained by varying PEG length and the dendron generation. Control over the extent of functionalization was investigated using fluorescence microscopy of the hydrogels after covalent attachment of a thiol-bearing fluorescent dye via thiol-ene “click” reaction. An increase in the amount of residual alkene groups within the hydrogel either by utilizing a higher generation dendron or by using a shorter polymeric component allows higher functionalization.

**Figure 21:**
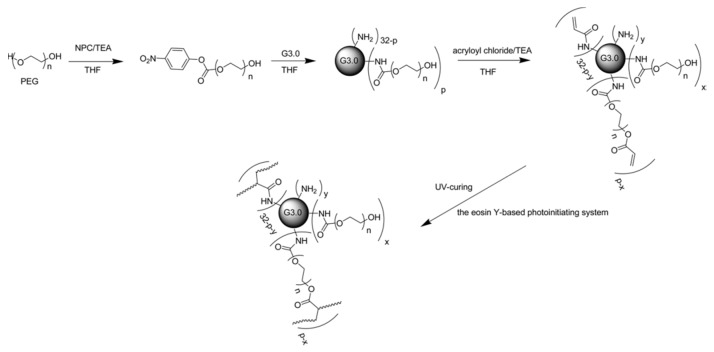
Synthesis of the photoreactive poly(amido amine) (PAMAM) dendron PEG conjugate and photoinitiated crosslinking for hydrogel formation. Reprinted with permission from [49], Copyright © 2010 American Chemical Society.

Fabrication of hydrogels for drug delivery and tissue engineering applications using photo-curable polymer conjugates of PAMAM dendrimers was reported by Yang and co-workers (Figure 21) [49]. They coupled the G3 PAMAM dendrimer with PEG chains of different lengths after the activation of PEG diols in the presence of 4-nitrophenyl chloroformate (NPC) and triethylamine (TEA). Photoreactive dendrimer polymer conjugates were obtained via attachment of acryloyl groups to residual amine groups of the PAMAM dendrimer and the hydroxyl group of PEG chains. An eosin-Y-based photo-initiating system was used to obtain a completely crosslinked network. In this study, they reported that the degree of PEGylation, PEG chain length and acrylate distribution determined the gelation and swelling parameters. The potential applications for tissue engineering and drug delivery were underlined due to the cyto-compatibility and structural characteristics of these hydrogels. However, in this strategy, it is not possible to obtain the selectively-acrylated distal end of the PEG chains due to the reactivity of acryloyl chloride towards free amine groups on the dendrimer periphery, during the acrylation of the PEGylated (PAMAM) dendrimer. As a result, efficient crosslinking was probably avoided due to possible shielding effect of PEG on the acrylate groups of the dendrimer surface.

To obtain efficient crosslinking, Yang and co-workers used a slightly different approach for the synthesis of photo-curable PEGylated (PAMAM) dendrimers [50]. They modified their previous approach by acrylating the PEG diol before conjugation with dendrimer. They used the G3 PAMAM dendrimer and PEG (12 kDa)-acrylate to obtain the PEGylated dendrimer and demonstrated a drug delivery system for the administration of two anti-glaucoma drugs. In this study, they prepared drug formulations by using the same initiation system to obtain hydrogels in the presence of one or both of the two anti-glaucoma drugs, brimonidine and timolol maleate in PBS. Because of the interior hydrophobic core of the PAMAM dendrimer and the hydrophilic nature of PEG, the hydrophobic drug brimonidine and the hydrophilic drug timolol maleate were both encapsulated within the hydrogel. They showed a dramatic increase in the water solubility of brimonidine, as well as intracellular uptake of the two anti-glaucoma drugs with sustained release by *in vitro* and *ex vivo* studies.

**Figure 22:**
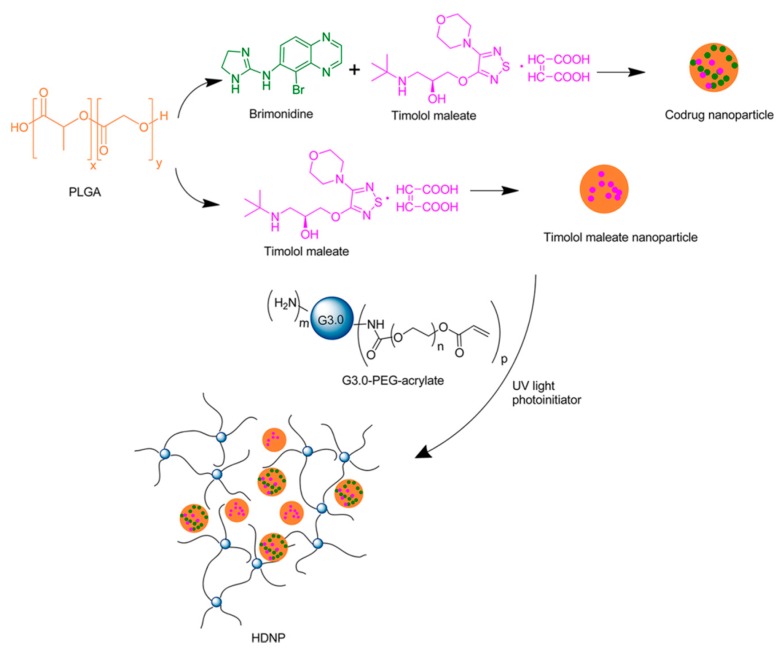
Formation of hydrogels incorporated with brimonidine- and timolol maleate-loaded nanoparticles (HDNP). Reprinted with permission from [51], Copyright © 2012 American Chemical Society. PLGA, poly(lactic-co-glycolic acid).

To further enhance the drug delivery system and to obtain better controlled drug release of the hydrogel dendrimer platform, Yang and co-workers formulated a hybrid dendrimer hydrogel/poly(lactic-co-glycolic acid) (PLGA) nanoparticle system with the two anti-glaucoma drugs (Figure 22) [51]. The dendrimer hydrogel drug formulation was prepared as mentioned above, and the nanoparticle drug formulation was prepared according to the literature procedure [52]. The PBS dispersion of drug-loaded PLGA nanoparticles was encapsulated into the dendrimer hydrogel during the crosslinking of the G3 PAMAM dendrimer PEG-acrylate conjugate. *In vitro* and *in vivo* results showed that this hybrid formulation provided combined drug delivery characteristics of its components and enhanced the drug release profiles of both nanoparticles and dendrimer hydrogels. Utilization of the dendrimer-based hydrogel also improved the mucoadhesive behavior of PLGA nanoparticles due to the amine groups on the dendrimer surface.

**Figure 23:**
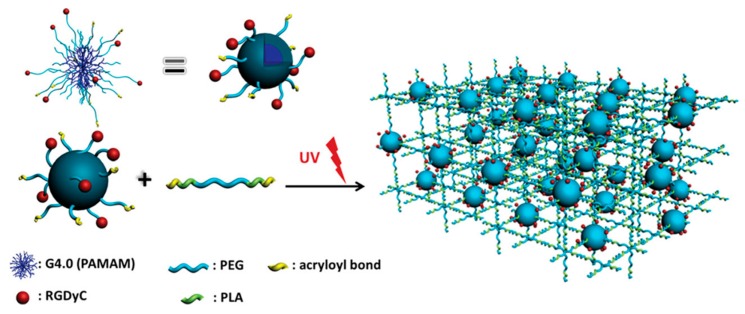
Schematic description of the hydrogels based on diacrylate-terminated poly(ethylene glycol)-*b*-poly(lactic acid) (PEG-PLA-DA) and the modified PAMAM dendrimer. From [53], Copyright © 2014 by John Wiley & Sons, Inc. Reprinted by permission of John Wiley & Sons, Inc. RGDyC, arginine-glycine-(aspartic acid)-(d-tyrosine)-cysteine.

A novel hydrogel was constructed via *in situ* photo-crosslinking for tissue engineering applications by Jia, Yang and co-workers [53]. Hydrogels were obtained through crosslinking of a diacrylate-terminated poly(ethylene glycol)-*b*-poly(lactic acid) (PEG-PLA-DA) triblock copolymer and a PAMAM dendrimer containing PEGs with acryloyl terminal groups. A G4 PAMAM dendrimer was modified to install acryloyl-terminated PEG segments and arginine-glycine-(aspartic acid)-(d-tyrosine)-cysteine (RGDyC) groups on the surface. Several hydrogels with varying ratios of the PEG-PLA-DA and modified PAMAM dendrimer were obtained under UV-irradiation (Figure 23). From *in vitro* experiments undertaken to evaluate the similarity of these hydrogels with the extracellular matrix, it was concluded that the presence of RGDyC enhanced cell attachment and proliferation.

**Figure 24:**
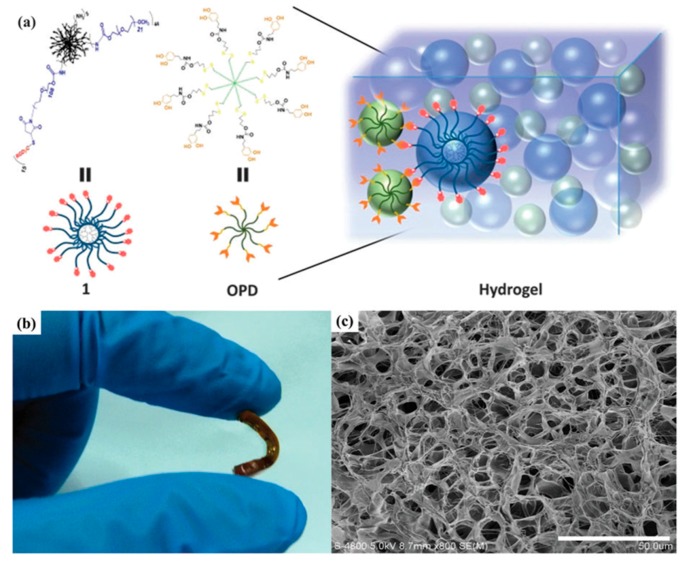
(**a**) Formation of hydrogel-based on the RGDyC-modified PEGylated PAMAM dendrimer and DOPA-terminated eight-armed PEG (OPD); the photograph (**b**) and SEM image (**c**) of the hydrogel. Reproduced from [54] with permission of The Royal Society of Chemistry.

Using a different approach, Jia, Yang and co-workers take advantage of oxidation-induced crosslinking for hydrogel formation using the dendrimer-polymer conjugate and hyper-branched PEG as building blocks (Figure 24) [54]. They used an alternative crosslinking strategy to form hydrogels using the RGDyC-modified PEGylated PAMAM dendrimer and DOPA-terminated eight-armed PEG for tissue engineering applications. Crosslinking was achieved by the oxidation-induced reaction in the presence of NaIO_4_ between DOPA and phenol groups of RGDyC. The aim was to benefit from the multifunctional structure of the PAMAM dendrimer to increase the crosslinking density, the strong adhesion property of DOPA to enhance the adhesion to tissue and the bioactivity of RGDyC to increase the success of the implanted material. This biodegradable hydrogel showed high mechanical strength and a low swelling ratio, which are desirable properties for bone tissue engineering applications. Additionally, *in vivo* studies (the mouse calvarial critical size defect model) demonstrated that this hydrogel enhanced the bone tissue formation when it was implanted. They underlined possible applications of this hydrogel system in other tissue engineering and clinical applications.

There are also several examples of physical hydrogel formation [55,56,57,58,59,60,61] using dendron polymer conjugates as building blocks. Namazi and Adeli synthesized dendron-linear polymer-dendrons (DLDs) composed of linear PEG and citric acid-based polyester dendrons for thermo-reversible hydrogel formation [55]. Hydrogels were prepared using solutions (2%, 4%, 6% and 8%, *w*/*v*) of DLDs in hot water followed by cooling below the transition temperature. To understand the host-guest behavior of DLDs, they prepared solutions of drug/DLD complexes in hot ethanol/water and obtained hydrogels in a similar manner. In another study to show the feasibility of these hydrogels as drug-delivery systems using several generations of PEG-based DLDs and formed drug/DLD complexes as described above [57].

**Figure 25:**
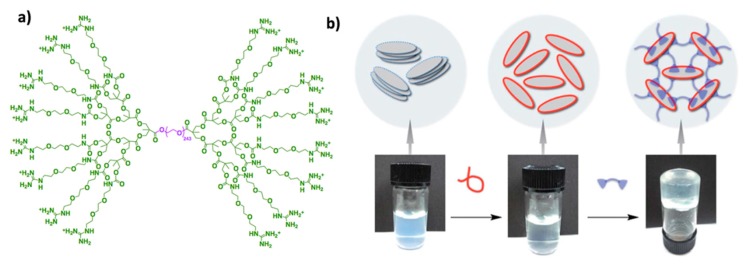
(**a**) DLD dendron-polymer conjugate with a linear PEG segment and G3 polyester dendrons displaying guanidinium ions; (**b**) dispersion of clay nanosheets by sodium polyacrylate and the addition of the positively-charged DLD with the G3 polyester dendron bearing guanidinium ions for crosslinking of clay nanosheets. Adapted by permission from Macmillan Publishers Ltd: Nature [57], Copyright © 2010.

A remarkable study of hydrogel formation was reported by Aida and co-workers, where they prepared physically-crosslinked hydrogels using guanidinium ion-terminated DLDs and clay nanosheets (CNS) [57]. They prepared the DLDs using a linear PEG segment and G1, G2 and G3 polyester dendrons displaying guanidinium ions. Clay nanosheets were dispersed using sodium polyacrylate prior to the hydrogel formation (Figure 25). The strong physical interaction between dispersed clay nanosheets and guanidinium ions on the periphery of the DLDs resulted in moldable hydrogels with great mechanical and self-healing properties compared to hydrogels prepared by conventional approaches, such as polymeric ions [58,59]. They tested the stability and activity of myoglobin inside these hydrogels and demonstrated the compatibility of these networks with biological applications.

**Figure 26:**
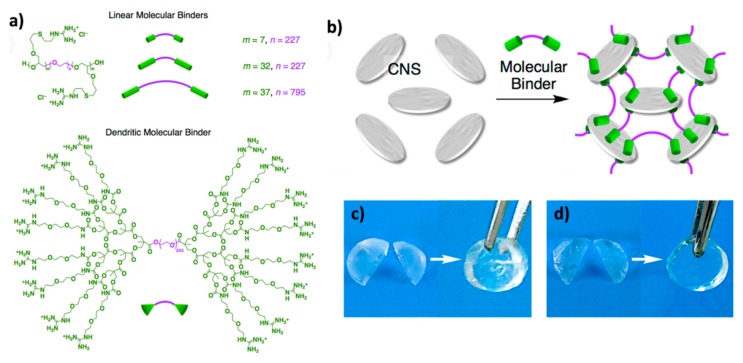
(**a**) Linear and dendritic molecular binders; (**b**) the addition of linear dendritic binder to exfoliated clay nanosheets for hydrogel formation. Self-repairing abilities of hydrogels formed by linear (**c**) and dendritic (**d**) molecular binders. Adapted with permission from [60], Copyright © 2013 American Chemical Society.

Although these dendritic molecular binders have various advantages to prepare aqueous networks, because of the multi-step synthesis of these dendritic binders, practical challenges are present for applications that require a large scale. Aida and co-workers designed linear polymeric binders to overcome this drawback and compared their physical properties to the dendritic ones (Figure 26) [60]. Hydrogels were formed in the same manner as described above. They reported that the hydrogels prepared by linear binders showed similar physical properties as the ones prepared by dendritic binders with one exception: dendritic binders showed faster hydrogel formation compared to linear binders, probably due to multiple functionality.

A dendron-polymer conjugate with a different architecture was utilized by Seelbach and co-workers. They demonstrated a versatile approach for cell-based regenerative medicine applications using injectable and thermo-responsive hydrogel composed of hyaluronic acid and peptide-based dendron conjugate and a thermo-gelling unit [61]. To begin with, they conjugated four integrin-binding sequences, RGDS (Arg-Gly-Asp-Ser) and DGRS (Asp-Gly-Arg-Ser) peptides to mono-azide-bearing diethylenetriamine pentaacetic acid with four oligo (ethylene glycol) aminoethyl branches and obtained peptide-based dendrons. Thereafter, they covalently grafted these dendrons to propargylamine-derived hyaluronic acid using the CuAAC reaction. They used the poly(*N*-isopropylacrylamide) and hyaluronan conjugate as a thermo-gelling unit. The hydrogels were prepared by a combination of these two types of conjugates in aseptic conditions. Promising *in vitro* results in cell culture experiments performed with human mesenchymal stromal cells showed the feasibility of the utilization of these hydrogels in the field of musculoskeletal repair.

## 5. Conclusions

Well-defined dendrimers and dendrons, as well as their polymeric conjugates serve as versatile components in the fabrication of hydrogels. The multivalent and multifunctional display of groups, as well as their branched structures impart important attributes to these materials that make them promising candidates for intended applications. Recent advances in the synthesis of dendrimeric constructs are making these once difficult to obtain materials more accessible. Importantly, the scalability and availability of these materials will also provide an assurance for increased utilization of these attractive building blocks in the design and synthesis of novel functional hydrogels.
